# Content validation of an interprofessional learning video peer assessment tool

**DOI:** 10.1186/s12909-017-1099-5

**Published:** 2017-12-16

**Authors:** Gillian Nisbet, Christine Jorm, Chris Roberts, Christopher J. Gordon, Timothy F. Chen

**Affiliations:** 1 Faculty of Health Sciences, The University of Sydney, 75 East street, Lidcombe, NSW 2141 Australia; 2 Sydney Medical School, The University of Sydney, Camperdown, NSW 2006 Australia; 3 Sydney Nursing School, The University of Sydney, Camperdown, NSW 2006 Australia; 4 Faculty of Pharmacy, The University of Sydney, Camperdown, NSW 2006 Australia

**Keywords:** Interprofessional learning, Content validity, Video-based, Assessment, Healthcare professional education

## Abstract

**Background:**

Large scale models of interprofessional learning (IPL) where outcomes are assessed are rare within health professional curricula. To date, there is sparse research describing robust assessment strategies to support such activities. We describe the development of an IPL assessment task based on peer rating of a student generated video evidencing collaborative interprofessional practice. We provide content validation evidence of an assessment rubric in the context of large scale IPL.

**Methods:**

Two established approaches to scale development in an educational setting were combined. A literature review was undertaken to develop a conceptual model of the relevant domains and issues pertaining to assessment of student generated videos within IPL. Starting with a prototype rubric developed from the literature, a series of staff and student workshops were undertaken to integrate expert opinion and user perspectives. Participants assessed five-minute videos produced in a prior pilot IPL activity. Outcomes from each workshop informed the next version of the rubric until agreement was reached on anchoring statements and criteria. At this point the rubric was declared fit to be used in the upcoming mandatory large scale IPL activity.

**Results:**

The assessment rubric consisted of four domains: patient issues, interprofessional negotiation; interprofessional management plan in action; and effective use of video medium to engage audience. The first three domains reflected topic content relevant to the underlying construct of interprofessional collaborative practice. The fourth domain was consistent with the broader video assessment literature calling for greater emphasis on creativity in education.

**Conclusions:**

We have provided evidence for the content validity of a video-based peer assessment task portraying interprofessional collaborative practice in the context of large-scale IPL activities for healthcare professional students. Further research is needed to establish the reliability of such a scale.

## Background

Interprofessional collaborative practice is considered fundamental for the delivery of safe, effective and efficient healthcare [1, 2]. The accreditation of many health professional courses requires universities to demonstrate how and where in curricula interprofessional learning (IPL) occurs to prepare graduates for the workplace [3]. While there is growing research evaluating small boutique IPL programs, reports of large-scale models with mandatory participation, where outcomes are assessed and graded, are rare within health professional curricula. Barriers to scalability and sustainability of IPL are well documented and include timetabling constraints, rigid curriculum structures, and limited faculty support [3–5]. The focus of this research addresses the gap in the IPL literature concerning evidence of sufficiently robust assessments to provide meaningful outcome measures of student achievement in large scale IPL. In prior work, we have demonstrated the feasibility and acceptability of an assessed large scale IPL activity (1220 students) that overcame many of the documented barriers to IPL [6]. In this paper we provide further detail of the assessment strategy which required peer assessment of videos produced by interprofessional student groups. The aim of this paper is to provide evidence of the content validity for a peer assessment rubric used in large-scale IPL activities. Content validity is defined as the extent to which the assessment covers the relevant content domain and whether it is at the right level of cognitive complexity [7].

### Critical appraisal of the literature

By critically appraising the literature, and based on our experience of IPL and its assessment, we developed a conceptual model of the relevant domains and issues pertaining to assessment of student generated videos within large scale IPL. This required investigation of the use of video projects as an assessable learning task within higher education, the required interprofessional competencies for health students and their assessment, and an assessment framework that did not rely on a heavy load for academics.

### Video-based assessment

Video-based project tasks have been implemented in a variety of higher education contexts where students are required to engage with novel concepts, challenge current thinking and/or influence change. Examples are found in foreign language teaching [8], political sciences [9], geography [10], science [11–13], medicine [14], dentistry [15], nursing [16], health promotion [17, 18], teacher education [19] and communication and ethics teaching [20].

While group project-based learning is not new, the ubiquity and affordability of video recording devices has created opportunities to use this technology. The pedagogical benefits of video-based projects are consistent with those documented for group learning [21, 22] including enhanced critical thinking and problem-solving, improved communication skills and socialisation to working collaboratively [11, 13, 18, 23].

Despite the growing use of video projects in learning and teaching, approaches to assessment of video products are less well-developed. Of the studies that have included assessment, criteria tend to focus on adequacy of topic content coverage, communication of the key message, creativity and the technical quality of the video product. For example, Lehman and colleagues [20] in their video project exploring ethical dilemmas around professional codes of conduct, emphasized the relevance and complexity of the ethical dilemma being presented, ability to engage the viewer, and video production quality in their assessment rubric. In a community engagement project where nursing students explored injury or violence prevention within a community, videos were judged on their ability to communicate a positive message, creativity, topic coverage; and video and audio quality [24]. In a project preparing education students for classroom diversity, Hall and Hudson [25] provided a rubric featuring video technical proficiency, ability to convey a message and knowledge of topic. In contrast, Haines and colleagues [18], in their project to promote the pharmacist’s role in public health, focussed mainly on topic content when assessing videos but did not provide the rubric. This is not uncommon in the literature (e.g. [8, 11, 24]). Studies that do include rubrics rarely include detail on the rubric development process used to establish their quality, this is a recognised deficiency in education literature [26].

### Assessment in IPL

Apart from our own work [6, 27], we were unable to locate any IPL studies describing the use of student-generated group video projects as a learning and teaching activity. Hence we did not locate any assessment tools specifically related to IPL video assessment. Tools are available to assess interprofessional practice competencies within the clinical setting (e.g. [28, 29, 30]) but these are not directly transferable to a group video task. However, interprofessional competency statements can be used to inform academic content of an IPL assessment rubric. Thistlethwaite and colleagues [31] conducted an extensive review of learning outcomes and competencies associated with interprofessional education programs. Learning outcomes were themed under eight headings: teamwork, roles/responsibilities, communication, learning/reflection, the patient and ethics/attitudes. Their subsequent work identified competency frameworks which incorporated many of these outcomes [32].

Arguably the most rigorously developed interprofessional competency frameworks in terms of national consultation are from Canada [33], the US [34, 35] and Australia [36]. The Canadian framework identified six competency domains for interprofessional collaboration: role clarification, team functioning, conflict resolution, collaborative leadership, interprofessional communication and patient/ client, family, community centred care [33]. Similarly, a US expert panel on interprofessional education (IPEC) included teamwork, communication, ethical practice, and roles and responsibilities in their framework [34, 35]. However, a patient, family and community focus informed all competencies in this framework, rather than it being a separate domain. In contrast to the US and Canadian frameworks, the Australian framework developed by O’Keefe and colleagues [36] provides a *single* competency statement for each of eight domains rather than the many identified in the other two frameworks. One domain is quite global and practical: the ability to plan patient/client care goals and priorities with other health professionals, possibly the crux of what needs to be demonstrated.

### Peer assessment

We drew on the Assessment 2020 framework [37], which advocates seven principles to improve assessment: engaging students, providing authentic activities, involving students in assessment design, developing learning and judgement, working with peers, giving and receiving feedback, and learning in the workplace. Peer assessment refers to processes which require students to ‘provide either feedback or grades (or both) to their peers on a product, process, or performance, based on the criteria of excellence for that product or event’ [38]. Within the higher education arena, peer assessment is a recognised learning and teaching strategy to develop student skills in giving and receiving feedback, reflective practice and accountability for personal learning [39–41]. Because students have spent considerable time producing their own work, when assessing others’ work within the same topic area, they begin to compare and contrast both pieces of work, invariably noting ways in which their own work could be improved [42], aiding student self-assessment and reflection [43]. Stone [44] suggested that peer assessment, in the context of IPL encourages cooperative learning, and should be weighted accordingly to signify its importance in the learning process. However, positive attitudes to peer assessment, depend on clear assessment criteria and rubrics [45]. Peer assessment is viewed negatively when students feel ill-prepared, if it is seen as a way of alleviating tutor marking responsibilities, or if there are concerns over its reliability [46].

In this context, we asked the research question ‘What are the elements which are important to students and staff in assessing a large scale video-based learning activity portraying interprofessional collaborative practice?’

## Method

### Assessment context

Prior to the large scale implementation described in this research, we had previously piloted a voluntary video assessment task in 2014, in part to address timetabling issues commonly associated with developing large scale IPL activities [27]. The pilot used an educational model adapted from the ‘Health Care Team Challenge’ [47, 48], where student teams (*n* = 77 students in 13 teams) were tasked with developing and presenting a management plan for a patient with a complex health condition. In the pilot, instead of preparing a traditional face-to-face presentation, student teams were asked to submit a five-minute video demonstrating their interprofessional management plan. These videos were assessed by the authors using a pilot video assessment rubric developed by the authors from the video assessment and IPL literature. The following year (2015) we extended the IPL activity and its assessment to the whole cohort of students (*n* = 1220 students in 208 teams). We acknowledged that the feasibility and sustainability would be severely compromised if we required over 200 academic markers who would require training and orientation. This was a key consideration in including the *peer* assessment of the student generated videos. A second important reason given our socio-cultural approach [37] was to capitalize on the known educational benefits of peer assessment. In order to assure the robustness of the video based assessment when marked by peers we were required to provide further evidence of its validity.

The 2015 IPL activity involved students working in interprofessional teams to develop a management plan for a patient case scenario. Student teams produced a 5 min video depicting an interprofessional approach as well as a one-page evidence-based management plan for the purpose of assuring the clinical content of the cases (not otherwise considered in this paper). Teams had 48 h to complete and submit these two assessment tasks. Using the assessment rubric described in this paper, students individually assessed two videos from teams that had completed the same case. Video ratings were submitted online via the University Learning Management System. Faculty assessed the written management plan.

### Content validation process

Evidence of content validity ensures educators and researchers are confident an assessment tool measures the constructs it is intended to measure [49]. We used two established models of scale development in an educational setting which draw on the available literature, expert opinion, and user perspectives [50, 51]. Together, they provide three important principles: the validation process should be guided by the tool’s intended function; established with the population using the assessment; and involve content experts [49–51].

We chose a rubric format as this format is commonly used within higher education and is generally positively accepted by students [26]. Rubrics comprise criteria to be evaluated, with unambiguous quality definitions explaining what a student must do to meet each level of achievement, and a scoring scale (e.g. unsatisfactory, satisfactory, good, excellent) [52].

As outlined by Brod et al. [50], a prototype IPL video peer assessment rubric which had been developed in the pilot of 2014 [27] was further developed by the authors from a more theoretically informed appraisal of the existing literature described earlier in this paper to develop a conceptual model which included relevant domains, and appropriate criteria and standards pertaining to assessment of student generated videos within IPL. We then subjected the rubric to the steps of a validation process outlined by Haynes et al. [51] to assure the content validity of the rubric prior to use in the large scale 2015 activity, with staff and students. This included developing consensus on the relevance of the domains being assessed; examining the proportional representation of items (criteria) so that they reflected the relative importance of the facets of the construct being assessed; and gaining experience of the tool by observing judgements of authentic student videos from the 2014 pilot in the validation process. Consistent with the validation process outlined by Brod and colleagues [50], we adopted a qualitative approach to capture the depth of discussion with participants.

### Participants

We used a purposive sampling technique [53], recruiting academic staff with an interest and/or experience in IPL and senior year students (year 3 or 4 of an undergraduate degree program or final year post graduate program) from health faculties within the university. Ethical approval for the study was granted by the University of Sydney Human Research Ethics Committee (Protocol number: 2015/320).

### Data generation

Students and staff from exercise physiology, nursing, occupational therapy, pharmacy, physiotherapy, and speech pathology were recruited for the content validation exercise. We conducted four workshops (two student and two staff workshops) in which 11 students and 8 staff participated (total *n* = 19). Workshops were facilitated by two of the authors (CJ and GN). The objectives of the workshops were to confirm items within the rubric were clear and understandable, that the important elements of interprofessional collaborative practice were fairly and appropriately represented within the rubric, and determine the levels of expectation on the quality of the student produced video.

All participants were asked to complete an IPL video peer assessment rubric for each of the three videos that they were shown. These videos had been produced as part of the previous year’s pilot IPL activity [27]. The videos had been selected to ensure they varied in technical quality, interprofessional content coverage, and ability to creatively communicate a message to a chosen audience. One video included a song depicting the patient’s experiences within the healthcare system (highly creative communication messaging; good content but low technical video quality); the second had a commercial focus (good messaging; high technical quality; somewhat superficial content); the other used a sporting analogy and demonstrated fair messaging, good content depth, and fair technical content. Participants were encouraged to write down examples from the videos that influenced their marking as well as comments related to their initial judgments of the video. Participants’ assessment marks for each criterion were compared and differences were used to invite participants to explain and justify their thinking. Discussion included description of how each participant interpreted the criteria, if/ how initial judgments of the video were captured within the rubric, additional criteria required, and suggested changes to wording to better reflect what was being assessed. The assessment marks given by participants were only used to generate discussion and were not analyzed further. Detailed field notes by the two authors were taken at each workshop. We also audio-recorded and transcribed all workshop discussions.

### Data analysis

Refinement of rubric clarity and relevance followed an iterative process whereby findings from the previous workshop informed the next workshops. Immediately following each workshop, two members of the research team (CJ and GN) reviewed the rubric reflecting on the workshop discussion, participant’s written notes, especially their suggested wording changes and/or additional criteria. Points of contention, confusion and/ or new meaning were identified and discussed by the researchers. Where agreement was reached amongst the researchers, changes were made to the rubric content to reflect new understanding and this new version used in subsequent workshops. Where agreement could not be reached, these points of contention or uncertainty were taken to the next workshop for further consideration. This process occurred until agreement was reached on anchoring statements and criteria by the researchers and scoring became consistent between participants. Following completion of all workshops, transcripts were analyzed deductively against the anchoring statements and criteria identified from the workshops. Using principles of framework analysis [54], each transcript was interrogated for supporting evidence and this mapped against the relevant domain of the rubric. Researcher field notes and participant written notes were reviewed against the rubric as a final triangulation of the data.

## Results

The peer assessment of a student generated video depicting interprofessional collaborative practice around a complex patient case rubric is given in Table 1.

**Table 1:** IPL Video Assessment Finalised Rubric (from Jorm et al., 2016)

	Unsatisfactory 1	Satisfactory 2	Good 3	Excellent 4
Patient issues	Issues faced by the patient and family not evident.	Describes the major issues faced by the patient and family.	Depicts appreciation of depth and/or breadth of issues faced by the patient and family.	Depicts considerable appreciation of depth and breadth of issues faced by the patient and family.
Interprofessional negotiation	Does not display negotiation, shared goal setting and shared decision making.	Shows limited appreciation of negotiation, shared goal setting and shared decision making.	Shows appreciation of negotiation, shared goal setting and shared decision making.	Sophisticated approach to negotiation, shared goal setting and shared decision making.
Interprofessional management plan in action	Management plan not evident in video.	Video depicts limited evidence of management plan in action.	Video depicts good evidence of management plan in action.	Strong depiction of co-ordinated and well executed interprofessional care.
Effective use of video medium to engage audience	Poor use of video medium.	Appropriate use of video medium.	Engaging.	Highly engaging and memorable.

It consists of a global rating and four domains: patient issues; interprofessional negotiation; interprofessional management plan in action; and effective use of video medium to engage an audience. A Likert scale rated the standard for each criterion from 1 = poor, 2 = satisfactory 3 = good to 4 = excellent. Descriptors are provided for each criterion and standard. We now present the data from interviews and field notes informing the validation process for each domain.

### Domain 1: Patient issues

This domain captured a central feature of interprofessional practice identified within the literature; a respect for the patient and family’s experiences and ability to view the situation through the lens of the patient and family [33, 34]. Our field notes illustrated how we had asked early in the validation process:

‘does the rubric emphasise the importance of patient-centredness/ patient perspective enough?’ (Workshop 2- field notes).

Participants in our study articulated this in various ways. For some, it encompassed demonstrating an appreciation of how the patient and family were feeling. For others, it included a sense that the patient had a say in the care process. Participants were able to recognise student engagement with a holistic approach to care, not merely focussing on the disciplinary clinical knowledge around a clinical diagnosis.

‘It was very personal. You actually delved into the person’s life instead of, you know, “this guy has a traumatic brain injury”. (Workshop 3 – student).

Equally, participants could identify when a patient’s perspective was poorly depicted, for example noting an example where the IPL team ‘didn’t even consult the client.’ (Workshop 1 – students). For others it was an issue of lack of student engagement with the patient as a person and not simply a clinical case.

‘[They] kept treating him like he wasn’t there. They didn’t engage him in the care process’. (Workshop 2 – staff).

Likewise, participants could identify when the perspectives of the IPL team was centred around the patient’s needs rather than around their disciplinary contributions to patient care.

‘So you see his issues from his point of view instead of all the healthcare professionals’. (Workshop 3 – students).

### Domain 2: Interprofessional negotiation

This domain captured a key feature of effective interprofessional practice – the ability to negotiate with other health professionals in problem solving for the patient [33, 36]. Professional priorities will always differ and knowledge of the potential contributions of other professionals is always incomplete. Participants considered negotiation to be fundamental to team collaborative decision-making, managing conflict, setting priorities and goal setting. As one participant noted:

‘If you’re going to share a decision, you have to negotiate too’. (Workshop 3 – students).

Participants were able to identify those videos that depicted a sophisticated understanding of interprofessional negotiation where a student team was able discuss patient problem priorities initially from a uni-professional perspective, and then prioritise the management around the needs of the patient.

‘Yeah, [I thought the first] video, that was the best example of that [negotiation] where they – I think it was the pharmacist and the doctor were talking at the beginning and then the case manager said no, let’s have a look at it from this angle.. . And the OT went hang on a minute.. . . They all got to chip in their priority and come to an agreement’. (Workshop 4 – staff).

Likewise, participants could identify when team collaborative decision-making was less evident. For example a participant noted how an IPL team separately reported their management from a disciplinary perspective, based on information transfer between at the most two disciplines within the negotiation.

‘.. . there was no discussion when they were all there I don’t think, was there?. . . ‘I don’t think they demonstrated how to share decision-making as a group’. They had a lot of two-way which was really good, the doctor referring to a radiographer, the nurse doing the home visit and then the – and saying she’d refer to the physiotherapist, but it was - do you know what I mean, there was lots of pairs rather than a team focus’. (Workshop 2 – staff).

While keen to see evidence of collaborative negotiation, the focus group members rejected teamwork as a specific domain to be assessed within the rubric.. As one participant explained, ‘.. . you can have a really great [team] presentation and then four out of five [students] were the ones that participated’ (Workshop 1 – students). In other words, the composite of team members in a presentation to assessors may be quite different to the internal teamwork dynamics. The participant made the analogy to patient care teams:

‘.. . if you’re working as a whole team [and] then there is a breakdown, but as professional, yeah, so you try and hide that because you want to present it as a. .. [unified team to the patient]’. (Workshop 1 – students).

It was acknowledged from practical experience that a skilled interprofessional team was likely to mask where a specific individual failed to contribute in teamwork. It seemed reasonable that a student team should do the same. Therefore actual teamwork cannot be marked in a video – only the depiction of teamwork.

### Domain 3: Interprofessional management plan in action

This domain captured the practicalities of interprofessional care including the coordination required for a well-executed interprofessional management plan [28, 36, 55]. Participants were able to differentiate videos that depicted individuals working *alongside* each other ‘.. . it was mostly the individual talking on their own about what their goal was.’ (Workshop 3 – students), from those that depicted an in-depth understanding and appreciation of the importance of working *together* around the patient:

‘That the interprofessionality necessarily invokes all of everyone’s caseloads. It’s about how they worked with each other around one person’. (Workshop 4 – staff).

They were also able to discern between videos that depicted a more sophisticated understanding of the impact of well-coordinated collaborative interprofessional care on patient care:

‘It’s implied in the outcome. We’ve seen what happens if you don’t coordinate: here’s what happens if you do coordinate even if we don’t show you the coordination.. .. In the way that they set up the storyline, they implied that it had to be coordinated to get that [outcome]. So they were showing an appreciation of the coordination. .. the impact of good coordination’. (Workshop 4 – staff).

Equally participants were able to discern when teams had addressed this domain superficially, even to the point of giving no marks for management planning.

‘I almost gave it a one but then I went, oh, hang on, a one is none and the Exercise Physiologist and Physio threw a ball together [to depict interaction] so it’s a two. .. but it [management plan] was pretty much non-existent’. (Workshop 4 – staff).

Whilst this domain called for coordination and a well-executed interprofessional management plan, participants acknowledged the reality that delivery of an interprofessional management plan can be difficult. However, by working together, teams were able to demonstrate the ways in which issues could be overcome. This was illustrated when discussing goal setting by the student teams:

‘It doesn’t happen, like you’re always going to have well “my goals are more important, no my goals are more important”. Although the video might have been poor [quality], they show that it’s not always perfect [IP practice]. That you need to work together as a team to overcome those issues’. (Workshop 4 – staff).

### Domain 4: Effective use of video medium to engage audience

Whilst the previous three domains encompassed the interprofessional literature, this domain tapped into the broader video assessment literature to include creativity. The literature suggests that video can be very creative and engage audiences to communicate a message more effectively than the written word [56]. Participant views on this aspect of the rubric were somewhat divided.

Participants could recognise a number of ways that engagement with an audience around IPL was achieved and hence could grade the videos accordingly. For example, videos that used sound, visual imagery and implied meaning were graded high:

‘It was the bluesy sad music when it wasn’t going well. .. using all the aspects they could have by putting a soundtrack to it and that’s what showed the difference between coordination and not coordination. I like it that they didn’t spell it out and go yes, we’re all working together, isn’t that lovely’. (Workshop 4 – staff).

Humour was also identified as being used in sophisticated ways to convey a message.

‘The humour was really good in that they were doing it from the patient’s point of view. No one’s listening to me, I’m going to crawl up in a little ball and you never saw him but you felt for him’. (Workshop 2 – staff).

However, some participants suggested a video that was highly creative in communicating could mask and confound other aspects of the assessment rubric, particularly criteria that focused on content stating they were ‘not convinced video has much substance. Quite vocal. Creativity masking substance’ (Workshop 4 – field notes).

A minority of participants viewed creativity as not a feature of the video but rather within the IPL process to indicate problem solving. This highlights the importance of assessors considering each criterion separately and reviewing the descriptors of that domain.

‘.. . I was looking at that video and I thought it really is engaging and it really does suck you in, but then when you’re looking at this [rubric], which is really I guess more the academic content.. . There wasn’t a lot of substance.’ (Workshop 4 – staff).

The inclusion of technical quality as a video assessment rubric criterion, as suggested by the literature [20, 24, 25] raised concern amongst participants and hence was not included in the final rubric. Students in particular were concerned with how *not to* disadvantage students with limited experience of the video medium. Use of video as an assessment task was rare for them. Hence, marking criteria needed to be cognisant of this. As one student explained:

‘… I would actually be a bit intimidated because I would consider myself a hard worker … I would feel very limited by my, literally by my video editing skills… I would get bogged down in, oh my gosh I’ve got to learn how to use this app, how to make this so that I can present it amazingly because I want the video to represent my knowledge and our group’s presentation of the idea and the assignment’. (Workshop 1 – students).

Fairness was also raised as an issue if technical quality was included as an assessed item: could people seek ‘outside’ help if they didn’t have the video skills? How would this be monitored? Could some teams gain unfair advantage by ‘.. . grabbing someone who is not even in the course and be like, “hey, come and edit my video for me”. (Workshop 3 – Students).

However, students were reluctant to totally discard marks for technical quality. They were concerned that if technical quality was weighted too low, students ‘.. . won’t make the effort on it’ (Workshop 3 – Students), reminding us of the old adage ‘assessment drives learning’. Workshop participants eventually recommended a minimum standard of video audibility and visual clarity be clearly outlined in overall task guidelines - rather than included in the rubric.

## Discussion

Findings form our content validation study have identified the major elements important to students and staff in assessing a large scale video-based learning activity portraying interprofessional collaborative practice. The assessment rubric was carefully derived from a critical appraisal of the IPL extant literature, existing group video-based assessment, contemporary assessment approaches and contexualized to the local setting through an iterative workshop process. Moreover, elements included in the rubric were observable behaviours that can be depicted readily and hence assessed within a video.

Our assessment rubric contained two domains that are consistent with the broader higher education video assessment literature: topic content coverage and creativity. The first three domains of our rubric, *patient issues*, *negotiation* and i*nterprofessional management plan in action* reflect topic content coverage; elements relevant to the underlying construct of interprofessional collaborative practice. These domains reflect the patient and family focus, conflict management and team-based practice of existing IPL frameworks (e.g. [28, 33, 34, 57]).

Topic content coverage required students to go beyond solely knowing about interprofessional practice to demonstrate *how* inteprofessional collaboration might look in practice. For example, our first domain *patient issues* required learners to empathize with what the patient and family were experiencing, reflecting the learner’s background knowledge of a patient-centred approach to care. Similarly our second domain identified a particular behaviour associated with conflict management; *negotiation*. Our third domain *interprofessional management plan in action* required learners to have a base knowledge of their own discipline to be able to incorporate, as appropriate, that perspective and practical solutions into the overall management plan.

Inclusion of the *Effective use of video medium to engage audience* domain within the assessment rubric (Domain 4) reflects the video assessment literature (e.g. [24]) and echoes calls for greater emphasis on creativity in education [58, 59]. Creativity does not feature prominently in the majority of healthcare curricula, and may be a foreign concept to students and faculty staff, resulting in divided views on its assessment. However, as Henriksen and colleagues argue, ‘creative thinking is essential for 21^st^ century success, as societal problems become more interdependent, global and complex’ [58]. Further, creativity has been espoused as essential for scientific inquiry and discovery and incorporated into teaching and learning activities using video [11]. If we want health professional students to develop problem solving skills, explore novel ideas and to engage with new technologies in their professional lives, we suggest curricula needs to incorporate activities and assessment that focus health professional students on creative elements.

In contrast to the video assessment literature, our validation process did not identify either communication or technical quality as separate domains. The ability to convey a clear message is a fundamental skill required for many aspects of interprofessional collaborative practice, for example, communicating with patients, negotiating with colleagues, or enacting a management plan. Our study participants were clear that more specific IPL measures should be the focus of the rubric and that communication skills were simply necessary to display good performance in the first three domains. This is in contrast to the Canadian [33] and US [34] frameworks which had interprofessional communication as separate domains, but consistent with O’Keefe et al. [36] where communication is implied throughout. Omission of technical quality from the finalalised rubric highlighting the participants’ unease at assessing students on something that is outside ‘usual practice’. This warrants further exploration as graduates will be constantly faced with new technologies and novel situations and will require the skills to adapt.

### Strengths and limitations

A strength of our study was inclusion of both academic staff and student perspectives in the validation of an assessment rubric. Academic staff needed to be confident in the grades awarded to students as this rubric was designed for both formative and summative assessment. Student input was needed as they are end users through the peer assessment process. Moreover, students presumably used the rubric when preparing their assessment task [60].

Although our finalised rubric contained a representative range of domains relevant to interprofessional education and was guided by the published literature, it was designed for a specific IPL activity. The rubric does not cover all aspects of collaborative practice identified in the literature. For example, leadership [33] and ethical practice [34] did not feature in the final rubric. This reflects the context of this particular assessment task and hence the importance participants placed on the competencies included in the rubric. Any single teaching and learning activity is unlikely to be able to deliver on every interprofessional competency. Therefore, our rubric may not be generalisable to other interprofessional educational contexts.

We acknowledge that some participants were challenged in separating assessment of content from creativity in the way the message was communicated to its intended audience. While this may reflect inexperience with creative technologies such as student-generated videos, this will be an important consideration and should be included in assessor training material and student orientation on the use of the rubric.

## Conclusion

We have provided content validation evidence for a novel interprofessional video-based peer assessment task in the context of large-scale IPL activities for healthcare professional students. This assessment task has overcome one of the barriers cited for the up-scaling and sustainability of IPL within the university context, namely inadequate validated IPL assessment approaches. Our research has enabled a previously voluntary IPL activity to be embedded as a mandatory assessed component of all health professional curricula. Future research using the rubric with a large student cohort will enable us to report on the reliability of the assessment tool.

The rubric cannot directly assess team dynamics. However, video production required students to work together. Future research should introduce feedback mechanisms for team members to assess team member’s contribution and performance against overall team performance. This would provide greater granularity for precise and fair individualised marks and feedback for all team members.

## Abbreviations

IPL: interprofessional learning
US: United States

## References

[1] Kohn LT, Corrigan JM, Donaldson MS (2000). To err is human: building a safer health system.

[2] World Health Organization. Framework for action on interprofessional education and collaborative practice. Geneva; 2010. Available from: http://www.who.int/hrh/resources/framework_action/en/21174039

[3] Nisbet G, Lee A, Kumar K, Thistlethwaite J, Dunston R. Interprofessional Health Education - A Literature Review - Overview of international and Australian developments in interprofessional health education (IPE). Learning and Teaching for Interprofessional Practice, Australia. Australian Learning and Teaching Council.; 2011.

[4] Lawlis TR, Anson J, Greenfield D (2014). Barriers and enablers that influence sustainable interprofessional education: a literature review. Journal of interprofessional care..

[5] Gilbert JHV (2005). Interprofessional learning and higher education structural barriers. Journal of Interprofessional Care..

[6] Jorm C, Nisbet G, Roberts C, Gordon C, Gentilcore S, Chen TF (2016). Using complexity theory to develop a student-directed interprofessional learning activity for 1220 healthcare students. BMC Med Educ.

[7] Lissitz RW, Samuelsen KA (2007). Suggested change in terminology and emphasis regarding validity and education. Educ Res.

[8] Nikitina L (2010). Video-making in the foreign language classroom: applying principles of constructivist pedagogy. Electronic Journal of Foreign Language Teaching.

[9] Florez-Morris M, Tafur I (2010). Using video production in political science courses as an instructional strategy for engaging students in active learning. Journal of Political Science Education.

[10] Graybill JK (2016). Teaching energy geographies via videography. J Geogr High Educ.

[11] Munakata M, Vaidya A (2015). Using project-and theme-based learning to encourage creativity in science. J Coll Sci Teach.

[12] Jensen M, Mattheis A, Johnson B (2012). Using student learning and development outcomes to evaluate a first-year undergraduate group video project. CBE-Life Sciences Education.

[13] Kabrhel JE. Debunking Pseudoscience: A Video Project To Promote Critical Thinking About Scientific Information in a General Chemistry Course. Integrating Information Literacy into the Chemistry Curriculum: ACS Publications. 2016:265–78.

[14] Kwan K, Wu C, Duffy D, Masterson J, Blair GK (2011). Lights, camera, surgery: a novel pilot project to engage medical students in the development of pediatric surgical learning resources. J Pediatr Surg.

[15] Omar H, Khan SA, Toh CG (2013). Structured student-generated videos for first-year students at a dental school in Malaysia. J Dent Educ.

[16] Pereira J, Echeazarra L, Sanz-Santamaría S, Gutiérrez J (2014). Student-generated online videos to develop cross-curricular and curricular competencies in nursing studies. Comput Hum Behav.

[17] Shuldman M, Tajik M (2010). The role of media/video production in non-media disciplines: the case of health promotion. Learning, Media and Technology.

[18] Haines SL, Van Amburgh JA (2010). A Vidcasting project to promote the pharmacist's role in public health. Am J Pharm Educ.

[19] Yang K-H (2014). Critical assessment of video production in teacher education: can video production foster community-engaged scholarship?. McGill Journal of Education/Revue des sciences de l'éducation de McGill.

[20] Lehman CM, DuFrene DD, Lehman MW (2010). YouTube video project: a “cool” way to learn communication ethics. Bus Commun Q.

[21] Cardellini L (2006). Fostering creative problem solving in chemistry through group work. Chemistry Education Research and Practice.

[22] Hansen RS (2006). Benefits and problems with student teams: suggestions for improving team projects. J Educ Bus.

[23] Hafner CA, Miller L (2011). Fostering learner autonomy in English for science: a collaborative digital video project in a technological learning environment. Language Learning & Technology.

[24] Krull H (2013). Video project brings new life to community engagement. J Nurs Educ.

[25] Hall L, Hudson R. Cross-curricular connections: video production in a K-8 teacher preparation program. Contemporary Issues in Technology and Teacher Education. 2006;6(3)

[26] Reddy YM, Andrade H (2010). A review of rubric use in higher education. Assessment & evaluation in higher education..

[27] Nisbet G, Gordon C, Jorm C, Chen T (2016). Influencing student attitudes through a student-directed Interprofessional learning activity: a pilot study. International journal of practice-based. Learn Health Soc Care.

[28] Brewer ML, Jones S (2013). An interprofessional practice capability framework focusing on safe, high-quality, client-centred health service. J Allied Health.

[29] Curran V, Hollett A, Casimiro LM, Mccarthy P, Banfield V, Hall P (2011). Development and validation of the interprofessional collaborator assessment rubric ((ICAR)). Journal of Interprofessional Care.

[30] Thistlethwaite J, Dallest K, Moran M, Dunston R, Roberts C, Eley D (2016). Introducing the individual teamwork observation and feedback tool (iTOFT): development and description of a new interprofessional teamwork measure. Journal of interprofessional care.

[31] Thistlethwaite J, Moran M (2010). Learning outcomes for interprofessional education (IPE): literature review and synthesis. Journal of Interprofessional Care..

[32] Thistlethwaite J, Forman D, Matthews LR, Rogers GD, Steketee C, Yassine T (2014). Competencies and frameworks in Interprofessional education: a comparative analysis. Acad Med.

[33] Canadian Interprofessional Health Collaborative. A national Interprofessional competency framework: University of British Columbia; 2010.

[34] Interprofessional education Collaborative. Core Competencies for Interprofessional Collaborative Practice: 2016 Update. Washington, DC, Interprofessional Education Collaborative. 2016.

[35] Schmitt M, Blue A, Aschenbrener CA, Viggiano TR (2011). Core competencies for interprofessional collaborative practice: reforming health care by transforming health professionals' education. Acad Med.

[36] O'Keefe M, Henderson A, Chick R. Defining a set of common interprofessional learning competencies for health profession students. Med Teacher. 2017;39(5):463–86. 10.1080/0142159X.2017.1300246 28332419

[37] Boud D, Dochy F. Assessment 2020. Seven propositions for assessment reform in. High Educ. 2010;

[38] Falchikov N. The place of peers in learning and assessment. In: Boud DF, N, editor. Rethinking assessment in higher education: learning for the longer term. London: Routledge; 2007. p. 128–143.

[39] Strijbos J-W, Sluijsmans D (2010). Unravelling peer assessment: methodological, functional, and conceptual developments. Learn Instr.

[40] Topping KJ (2009). Peer assessment. Theory Pract.

[41] Nicol D, Thomson A, Breslin C (2014). Rethinking feedback practices in higher education: a peer review perspective. Assessment & Evaluation in Higher Education..

[42] Green C (2013). Relative distancing: a grounded theory of how learners negotiate the interprofessional. Journal of interprofessional care..

[43] Miers ME, Clarke BA, Pollard KC, Rickaby CE, Thomas J, Turtle A (2007). Online interprofessional learning: the student experience. Journal of Interprofessional Care..

[44] Stone J (2010). Moving interprofessional learning forward through formal assessment. Med Educ.

[45] Andrade H, Du Y (2007). Student responses to criteria-referenced self-assessment. Assessment & evaluation in higher education.

[46] Davies P (2003). Closing the communications loop on the computerized peer-assessment of essays. Association for Learning Technology. Journal.

[47] Moran M, Boyce R, O'Neill K, Bainbridge L, Newton C (2007). The health care team challenge: extra-curricula engagement in inter-professional education (IPE). Focus on health professional education: a multi-disciplinary. Journal.

[48] Newton C, Bainbridge L, Ball VA, Wood VI (2013). Health care team challenges: an international review and research agenda. Journal of Interprofessional Care..

[49] Vogt DS, King DW, King LA (2004). Focus groups in psychological assessment: enhancing content validity by consulting members of the target population. Psychol Assess.

[50] Brod M, Tesler LE, Christensen TL (2009). Qualitative research and content validity: developing best practices based on science and experience. Qual Life Res.

[51] Haynes SN, Richard D, Kubany ES (1995). Content validity in psychological assessment: a functional approach to concepts and methods. Psychol Assess.

[52] Popham WJ (1997). What’s wrong-and what's right-with rubrics. Educ Leadersh.

[53] Cohen L, Manion L, Morrison K. Research methods in education. 5 ed. London: Routledge Falmer; 2005. 446 p.

[54] Gale NK, Heath G, Cameron E, Rashid S, Redwood S (2013). Using the framework method for the analysis of qualitative data in multi-disciplinary health research. BMC Med Res Methodol.

[55] Walsh CL, Gordon MF, Marshall M, Wilson F, Hunt T (2005). Interprofessional capability: a developing framework for interprofessional education. Nurse Educ Pract.

[56] Robin BR (2008). Digital storytelling: a powerful technology tool for the 21st century classroom. Theory Pract.

[57] Gum LF, Lloyd A, Lawn S, Richards JN, Lindemann I, Sweet L (2013). Developing an interprofessional capability framework for teaching healthcare students in a primary healthcare setting. Journal of interprofessional care..

[58] Henriksen D, Mishra P, Fisser P (2016). Infusing creativity and technology in 21st century education: a systemic view for change. Educational Technology & Society.

[59] Shaheen R (2010). Creativity and education. Creative Education.

[60] Grainger P, Christie M, Thomas G, Dole S, Heck D, Marshman M, et al. Improving the quality of assessment by using a community of practice to explore the optimal construction of assessment rubrics. Reflective Pract. 2017:1–13.

